# Crystal structure of 1-(4-bromo­phen­yl)but-3-yn-1-one

**DOI:** 10.1107/S205698902300508X

**Published:** 2023-06-13

**Authors:** Shaziyaparveen K. Siddiqui, C. V. Ramana, Rajesh G. Gonnade

**Affiliations:** a Division of Organic Synthesis, CSIR-National Chemical Laboratory, Dr. Homi, Bhabha Road, Pashan, Pune-411008, India; b Academy of Scientific and Innovative Research (AcSIR), Sector 19, Kamla Nehru Nagar, Ghaziabad, Uttar Pradesh 201002, India; c Physical & Materials Chemistry Division, CSIR-National Chemical Laboratory, Dr. Homi Bhabha Road, Pune-411008, India; Indian Institute of Science Education and Research Bhopal, India

**Keywords:** crystal structure, inter­molecular inter­actions, Hirshfeld surface analysis

## Abstract

The title compound, 1-(4-bromo­phen­yl)but-3-yn-1-one, C_10_H_7_BrO, crystallizes in the monoclinic space group *P*2_1_/*n* with one mol­ecule in the asymmetric unit. The structure displays a planar geometry. The crystal structure is consolidated by C—H⋯O hydrogen bonding and a short C=O⋯C≡C (acetyl­ene) contacts. Hirshfeld surface analysis indicates that H⋯H, C⋯H/H⋯C and H⋯Br/Br⋯H inter­actions play a more important role in consolidating the crystal structure compared to H⋯O/O⋯H and C⋯C contacts.

## Abstract 2

The title compound crystallizes in the monoclinic crystal system in the centrosymmetric space group *P*2_1_/*n*. The crystal structure features C—H⋯O hydrogen bonding and a short C=O⋯C≡C (acetyl­ene) contacts.

## 1. Chemical context

The title compound 1-(4-bromo­phen­yl)but-3-yn-1-one (**1**) was obtained as a side product during the synthesis of 5-(4-bromo­phen­yl)isoxazole-3-carb­oxy­lic acid (**2**) from the NaOH-mediated hydrolysis of ethyl 5-(4-bromo­phen­yl)isoxazole-3-carboxyl­ate (**3**). These aryl­isoxazole carb­oxy­lic acids have been identified as potential isosteres of aryl diketo acid in the design of novel HIV-1 integrase inhibitors (Zeng *et al.*, 2008 ▸). The presence of three distinct functional groups, viz. alkyne, bromo, and carbonyl, offers an intriguing opportunity to explore how inter­molecular inter­actions contribute to the cohesion of the crystal structure. 
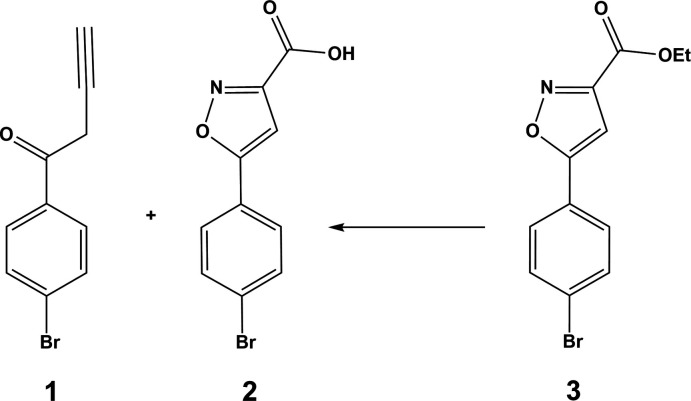


## 2. Structural commentary

**Figure 1:**
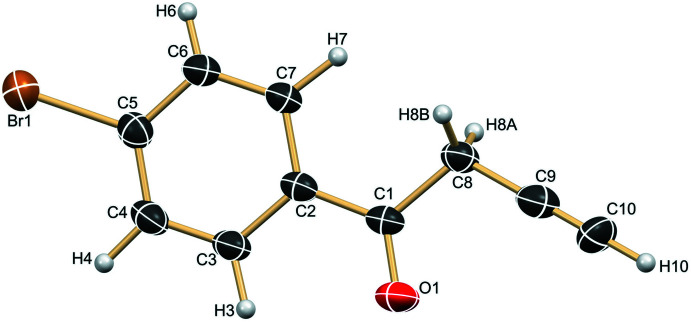
The asymmetric unit of **1** with the atom labelling. Displacement ellipsoids represent 30% probability levels.

The title compound crystallizes in the monoclinic *P*2_1_/*n* centrosymmetric space group with one mol­ecule of **1** in the asymmetric unit (Fig. 1 ▸). The structure displays a planar geometry [torsion angle C5—C1—C8—C9 = 175.4 (3)^o^, only the C5 atom of phenyl ring is considered and not the full fragment]. The phenyl ring makes a dihedral angle of 5.4 (2)° with the least-squares plane through the O1/C1/C8–C10 fragment.

## 3. Supra­molecular features

**Figure 2:**
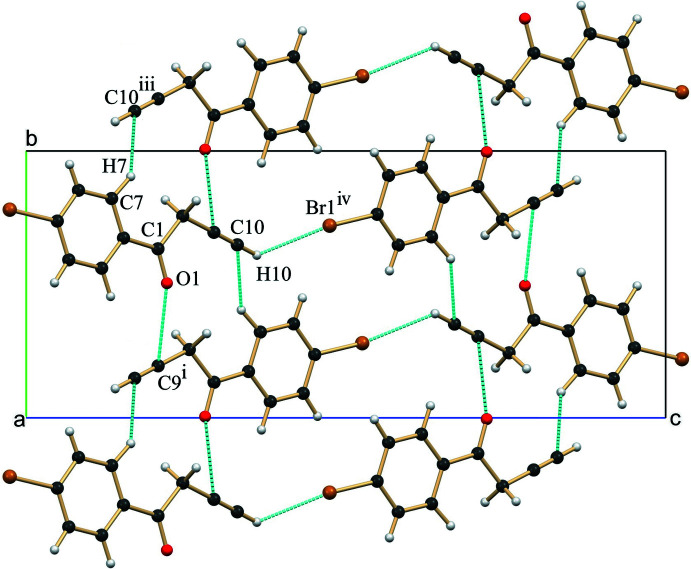
A view of the mol­ecular packing of **1** along the helical *b*-axis showing the association of closely linked mol­ecules by C=O⋯C≡C (acetyl­ene, C=O⋯π) and marginal C—H⋯π (π-cloud of acetyl­ene mol­ecules) contacts. Neighbouring helices along the longer *c* axis are linked by C—H⋯Br contacts. Symmetry codes as in Table 1 ▸.

**Figure 3:**
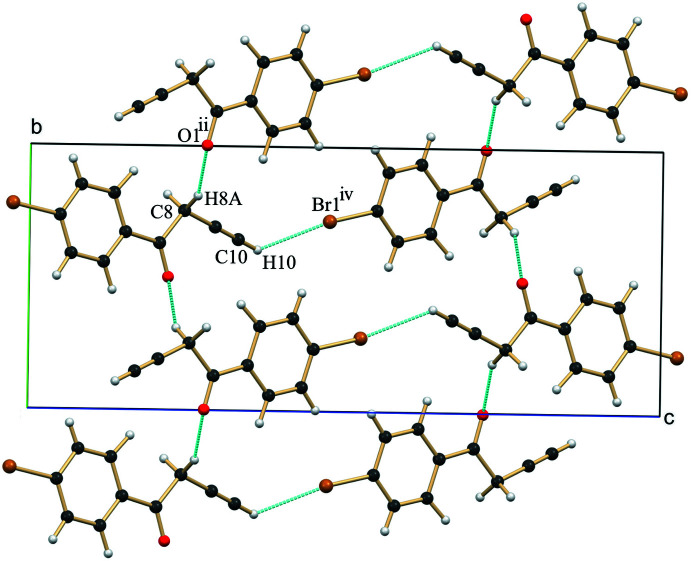
A view of the mol­ecular packing of **1** along the helical *b*-axis showing the association of closely linked mol­ecules by C—H⋯O contacts. Neighbouring helices along the longer *c* axis are linked by C—H⋯Br contacts. Symmetry codes as in Table 1 ▸.

In the crystal, the closely associated mol­ecules of **1** generate two different helical assemblies across the crystallographic 2_1_-screw axis (*b*-axis). The helical assembly generated using C1=O1⋯C9^i^≡C10^i^ (acetyl­ene, C=O⋯π) contacts [symmetry code: (i) −*x* + 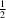 , *y* + 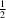 , −*z* + 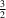 ] (Li *et al.*, 2019 ▸; Mooibroek, *et al.*, 2008 ▸) has a sheet structure (Fig. 2 ▸, Table 1 ▸), while the helical assembly created using C—H⋯O (C8—H8*A*⋯O1^ii^) contacts [symmetry code: (ii) −*x* + 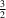 , *y* − 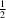 , −*z* + 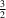 ] (Desiraju & Steiner, 2001 ▸) has a proper helical structure (Fig. 3 ▸, Table 1 ▸). The helical assembly created using the short C1=O1⋯C9≡C10 contacts is further supported by marginal C—H⋯π [symmetry code: (iii) −*x* + 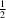 , *y* − 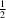 , −*z* + 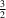 ] contacts involving the phenyl ring (C7—H7) and the π cloud of the acetyl­ene moiety. Both helices are inter­twined and form a two-dimensional sheet structure roughly along the *a*-axis direction. Along the longer *c*-axis, mol­ecules are loosely connected using weak C—H⋯Br (C10—H10⋯Br1^iv^ contacts [symmetry code: (iv) *x* − 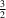 , −*y* + 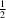 , *z* − 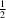 ] (van den Berg & Seddon, 2003 ▸), generating the extended assembly (Figs. 2 ▸ and 3 ▸, Table 1 ▸).

**Figure 4:**
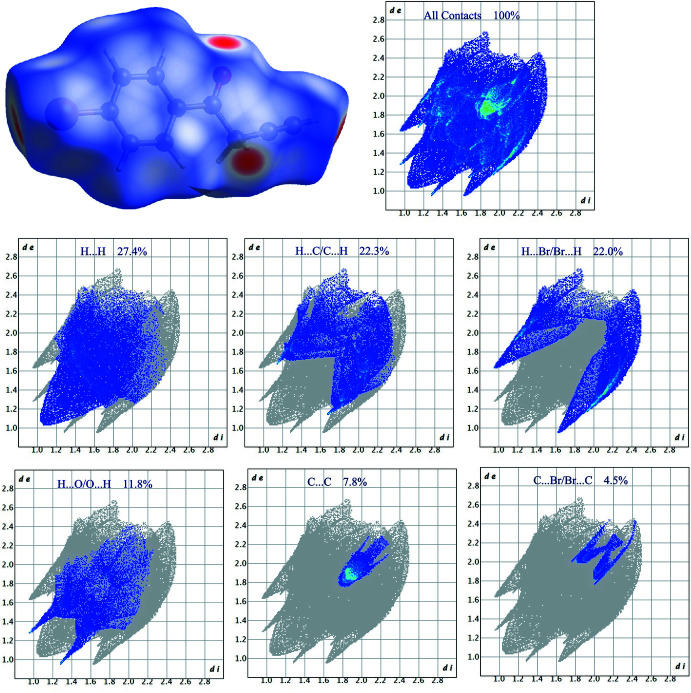
Three-dimensional Hirshfeld surfaces of compound **1** plotted over *d*_norm_ in the range −0.2760 to 0.9829 a.u., and Hirshfeld fingerprint plots for all contacts and those decomposed into H⋯H, H⋯C/C⋯H, H⋯Br/Br⋯H, H⋯O/O⋯H, C⋯C and C⋯Br/Br⋯C contacts. *d*_i_ and *d*_e_ denote the closest inter­nal and external distances (in Å) from a point on the surface.

In order to visualize and qu­antify inter­molecular inter­actions in **1**, a Hirshfeld surface analysis (Spackman & Jayatilaka, 2009 ▸) was performed using *Crystal Explorer 21.5* (Spackman *et al.*, 2021 ▸), and the associated two-dimensional fingerprint plots (McKinnon *et al.*, 2007 ▸) were generated. The Hirshfeld surfaces for the mol­ecule in **1** are shown in Fig. 4 ▸ in which the two-dimensional fingerprint plots of the most dominant contacts are also presented. H⋯H (27.4%), H⋯C/C⋯H (22.3%) and H⋯Br/Br⋯H (22.0%) contacts are responsible for the largest contributions to the Hirshfeld surface. Besides these contacts, H⋯O/O⋯H (11.8%) and C⋯C (7.8%) inter­actions contribute significantly to the total Hirshfeld surface. The contributions of further contacts are only minor and amount to C⋯Br/Br⋯C (4.5%) and C⋯O/O⋯C (3.6%).

## 4. Database survey

A survey of the Cambridge Structural Database (version 5.43, update 4, November 2022; Groom *et al.*, 2016 ▸) revealed that no crystal structure of compound **1** has been reported. Moreover, no crystal structure similar to that of compound **1** has been reported. However, focusing only on the 1-phenyl­but-3-yn-1-one unit yielded 24 hits with not much similarity with the title compound. The most similar structure with respect to compound **1** is 3-phenyl-2-(phenyl­ethyn­yl)-1*H*-inden-1-one (FEGDOO; Kumar *et al.*, 2022 ▸).

## 5. Synthesis and crystallization

A solution of methyl ester **3** (100 mg, 0.35 mmol) and 1*N* NaOH (3 mL) and methanol (3 mL) was heated to reflux for 3 h. After completion of the reaction as indicated by TLC, the reaction mixture was cooled to room temperature and neutralized with a solution of 3N HCl and then extracted with di­chloro­methane (3 × 10 mL). The combined organic layer was washed with brine and concentrated. The resulting crude was purified by column chromatography (30% ethyl acetate in petroleum ether) to afford the acid **2** (70 mg, 74% yield) and an alkyne, the title compound **1** (8 mg, 11% yield) as colourless solids. Colourless crystals of the title compound **1** suitable for single crystal X-ray diffraction analysis were obtained by slow evaporation of an ethanol solution.

## 6. Refinement

Crystal data, data collection and structure refinement details are summarized in Table 2 ▸. All H atoms (except the acetyl­ene H atom) were located in difference-Fourier map and refined isotropically. The acetyl­ene (—C≡C—H) H atom was placed in a geometrically idealized position using HFIX 163. It was constrained to ride on its parent atom, with *U*_iso_(H) = 1.2*U*_eq_(C) for acetyl­ene. The long C8—H8*A* distance [1.02 (4) Å] could be the result of its involvement in the directional C— H⋯O hydrogen-bond formation with O1.

## Supplementary Material

Crystal structure: contains datablock(s) I. DOI: 10.1107/S205698902300508X/dx2053sup1.cif

Structure factors: contains datablock(s) I. DOI: 10.1107/S205698902300508X/dx2053Isup2.hkl

Supporting information file. DOI: 10.1107/S205698902300508X/dx2053Isup3.cml

CCDC reference: 2268276

Additional supporting information:  crystallographic information; 3D view; checkCIF report

## Figures and Tables

**Table 1:** Hydrogen-bond geometry (Å, °)

*D*—H⋯*A*	*D*—H	H⋯*A*	*D*⋯*A*	*D*—H⋯*A*
C1—O1⋯C9^i^		3.13 (1)		154 (1)
C8—H8*A*⋯O1^ii^	1.02 (4)	2.31 (4)	3.259 (5)	156 (3)
C7—H7⋯C10^iii^	1.06 (3)	2.71 (4)	3.626 (5)	144 (3)
C10—H10⋯Br1^iv^	0.93	2.68	3.305 (5)	126

Symmetry codes: (i) 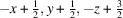 ; (ii) 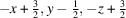 ; (iii) 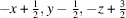 ; (iv) 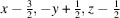 .

**Table 2:** Experimental details

Crystal data	
Chemical formula	C_10_H_7_BrO
*M*_r_	223.07
Crystal system, space group	Monoclinic, *P*2_1_/*n*
Temperature (K)	297
*a*, *b*, *c* (Å)	4.471 (2), 9.032 (4), 21.652 (11)
β (°)	92.252 (8)
*V* (Å^3^)	873.7 (7)
*Z*	4
Radiation type	Mo *K*α
μ (mm^−1^)	4.65
Crystal size (mm)	0.35 × 0.28 × 0.13

Data collection	
Diffractometer	Bruker SMART APEX
Absorption correction	Multi-scan (*SADABS*; Bruker 2016 ▸)
*T*_min_, *T*_max_	0.293, 0.583
No. of measured, independent and observed [*I* > 2σ(*I*)] reflections	4994, 1965, 1397
*R*_int_	0.043
(sin θ/λ)_max_ (Å^−1^)	0.664

Refinement	
*R*[*F* ^2^ > 2σ(*F* ^2^)], *wR*(*F* ^2^), *S*	0.048, 0.140, 1.02
No. of reflections	1965
No. of parameters	133
H-atom treatment	H atoms treated by a mixture of independent and constrained refinement
Δρ_max_, Δρ_min_ (e Å^−3^)	0.40, −0.62

Computer programs: *APEX3* and *SAINT-Plus* (Bruker, 2016 ▸), *SHELXS97* (Sheldrick, 2008 ▸), *SHELXL2014/7* (Sheldrick, 2015 ▸), *ORTEP-3 for Windows* (Farrugia, 2012 ▸), *Mercury* (Macrae *et al.*, 2020 ▸), *SHELXTL* (Sheldrick, 2008 ▸), *PLATON* (Spek, 2020 ▸) and *publCIF* (Westrip, 2010 ▸).

## supplementary crystallographic information

### Crystal data

**Table:**

C_10_H_7_BrO	*F*(000) = 440
*M**_r_* = 223.07	*D*_x_ = 1.696 Mg m^−^^3^
Monoclinic, *P*2_1_/*n*	Mo *K*α radiation, λ = 0.71073 Å
*a* = 4.471 (2) Å	Cell parameters from 1876 reflections
*b* = 9.032 (4) Å	θ = 2.4–24.9°
*c* = 21.652 (11) Å	µ = 4.65 mm^−^^1^
β = 92.252 (8)°	*T* = 297 K
*V* = 873.7 (7) Å^3^	Block, colourless
*Z* = 4	0.35 × 0.28 × 0.13 mm

### Data collection

**Table:**

Bruker SMART APEX diffractometer	1965 independent reflections
Radiation source: fine-focus sealed tube	1397 reflections with *I* > 2σ(*I*)
Graphite monochromator	*R*_int_ = 0.043
Phi and ω Scan scans	θ_max_ = 28.2°, θ_min_ = 2.4°
Absorption correction: multi-scan (SADABS; Bruker 2016)	*h* = −4→5
*T*_min_ = 0.293, *T*_max_ = 0.583	*k* = −11→11
4994 measured reflections	*l* = −27→27

### Refinement

**Table:**

Refinement on *F*^2^	Primary atom site location: structure-invariant direct methods
Least-squares matrix: full	Hydrogen site location: mixed
*R*[*F*^2^ > 2σ(*F*^2^)] = 0.048	H atoms treated by a mixture of independent and constrained refinement
*wR*(*F*^2^) = 0.140	*w* = 1/[σ^2^(*F*_o_^2^) + (0.0812*P*)^2^ + 0.1069*P*] where *P* = (*F*_o_^2^ + 2*F*_c_^2^)/3
*S* = 1.02	(Δ/σ)_max_ = 0.002
1965 reflections	Δρ_max_ = 0.40 e Å^−^^3^
133 parameters	Δρ_min_ = −0.62 e Å^−^^3^
0 restraints	

### Special details

**Table:**

Geometry. All esds (except the esd in the dihedral angle between two l.s. planes) are estimated using the full covariance matrix. The cell esds are taken into account individually in the estimation of esds in distances, angles and torsion angles; correlations between esds in cell parameters are only used when they are defined by crystal symmetry. An approximate (isotropic) treatment of cell esds is used for estimating esds involving l.s. planes.

### Fractional atomic coordinates and isotropic or equivalent isotropic displacement parameters (Å^2^)

**Table:**

	*x*	*y*	*z*	*U*_iso_*/*U*_eq_	
C1	0.5990 (7)	0.3666 (3)	0.79382 (16)	0.0508 (8)	
Br1	1.37959 (10)	0.22006 (5)	1.02549 (2)	0.0743 (2)	
C2	0.7972 (7)	0.3261 (3)	0.84766 (16)	0.0488 (7)	
C3	0.9384 (9)	0.4391 (4)	0.88181 (17)	0.0581 (9)	
H3	0.887 (9)	0.545 (5)	0.8679 (19)	0.080 (12)*	
C4	1.1186 (9)	0.4079 (4)	0.93323 (18)	0.0615 (9)	
H4	1.217 (9)	0.494 (5)	0.9555 (19)	0.077 (11)*	
C5	1.1578 (9)	0.2628 (4)	0.95122 (18)	0.0556 (8)	
C6	1.0233 (9)	0.1475 (4)	0.91808 (18)	0.0609 (9)	
H6	1.065 (9)	0.051 (5)	0.9319 (19)	0.070 (11)*	
C7	0.8439 (9)	0.1783 (4)	0.86619 (17)	0.0560 (8)	
H7	0.752 (8)	0.096 (4)	0.8364 (15)	0.054 (9)*	
C8	0.4544 (9)	0.2439 (4)	0.7551 (2)	0.0555 (9)	
H8B	0.342 (9)	0.184 (4)	0.7824 (18)	0.056 (10)*	
C9	0.2564 (10)	0.3048 (4)	0.7064 (2)	0.0666 (11)	
H8A	0.617 (10)	0.187 (5)	0.734 (2)	0.068 (12)*	
C10	0.1011 (10)	0.3516 (5)	0.6698 (2)	0.0783 (12)	
H10	−0.0283	0.3905	0.6393	0.094*	
O1	0.5514 (7)	0.4954 (2)	0.77985 (13)	0.0718 (8)	

### Atomic displacement parameters (Å^2^)

**Table:**

	*U*^11^	*U*^22^	*U*^33^	*U*^12^	*U*^13^	*U*^23^
C1	0.0553 (18)	0.0353 (15)	0.0622 (19)	−0.0025 (13)	0.0097 (15)	−0.0018 (14)
Br1	0.0827 (4)	0.0702 (3)	0.0688 (3)	−0.00322 (18)	−0.0116 (2)	−0.00504 (18)
C2	0.0542 (18)	0.0368 (14)	0.0563 (18)	−0.0031 (13)	0.0125 (15)	−0.0051 (14)
C3	0.076 (2)	0.0382 (16)	0.061 (2)	−0.0070 (15)	0.0057 (18)	−0.0033 (15)
C4	0.074 (2)	0.0484 (19)	0.062 (2)	−0.0152 (17)	0.0072 (18)	−0.0110 (17)
C5	0.060 (2)	0.0547 (19)	0.0524 (19)	−0.0042 (15)	0.0068 (15)	−0.0065 (16)
C6	0.077 (2)	0.0415 (18)	0.064 (2)	−0.0030 (17)	0.0016 (18)	−0.0003 (16)
C7	0.069 (2)	0.0386 (15)	0.060 (2)	−0.0030 (15)	0.0009 (17)	−0.0053 (15)
C8	0.057 (2)	0.0396 (15)	0.069 (2)	0.0016 (15)	−0.0034 (19)	−0.0029 (16)
C9	0.067 (2)	0.0464 (18)	0.087 (3)	−0.0048 (17)	0.002 (2)	−0.0019 (19)
C10	0.084 (3)	0.060 (2)	0.088 (3)	0.005 (2)	−0.032 (2)	0.017 (2)
O1	0.0875 (18)	0.0377 (12)	0.0895 (19)	0.0002 (12)	−0.0066 (16)	0.0024 (13)

### Geometric parameters (Å, º)

**Table:**

C1—O1	1.218 (4)	C5—C6	1.388 (5)
C1—C2	1.482 (5)	C6—C7	1.383 (5)
C1—C8	1.518 (5)	C6—H6	0.93 (4)
Br1—C5	1.895 (4)	C7—H7	1.06 (3)
C2—C3	1.397 (5)	C8—C9	1.458 (7)
C2—C7	1.407 (5)	C8—H8B	0.96 (4)
C3—C4	1.378 (6)	C8—H8A	1.02 (4)
C3—H3	1.03 (5)	C9—C10	1.117 (6)
C4—C5	1.377 (5)	C10—H10	0.9300
C4—H4	1.01 (5)		
			
O1—C1—C2	121.6 (3)	C7—C6—C5	119.7 (3)
O1—C1—C8	119.6 (3)	C7—C6—H6	123 (3)
C2—C1—C8	118.8 (3)	C5—C6—H6	117 (3)
C3—C2—C7	118.9 (3)	C6—C7—C2	119.8 (3)
C3—C2—C1	118.7 (3)	C6—C7—H7	123.5 (18)
C7—C2—C1	122.4 (3)	C2—C7—H7	116.4 (18)
C4—C3—C2	121.1 (3)	C9—C8—C1	110.9 (3)
C4—C3—H3	123 (2)	C9—C8—H8B	110 (2)
C2—C3—H3	116 (2)	C1—C8—H8B	107 (2)
C5—C4—C3	119.2 (3)	C9—C8—H8A	107 (2)
C5—C4—H4	123 (2)	C1—C8—H8A	109 (2)
C3—C4—H4	117 (3)	H8B—C8—H8A	113 (3)
C4—C5—C6	121.3 (4)	C10—C9—C8	178.8 (6)
C4—C5—Br1	119.4 (3)	C9—C10—H10	180.0
C6—C5—Br1	119.2 (3)		
			
O1—C1—C2—C3	−1.9 (5)	C3—C4—C5—Br1	−175.1 (3)
C8—C1—C2—C3	177.7 (3)	C4—C5—C6—C7	−0.6 (6)
O1—C1—C2—C7	177.0 (3)	Br1—C5—C6—C7	175.5 (3)
C8—C1—C2—C7	−3.4 (5)	C5—C6—C7—C2	−0.5 (6)
C7—C2—C3—C4	−0.7 (5)	C3—C2—C7—C6	1.1 (6)
C1—C2—C3—C4	178.3 (3)	C1—C2—C7—C6	−177.8 (3)
C2—C3—C4—C5	−0.4 (6)	O1—C1—C8—C9	−3.3 (6)
C3—C4—C5—C6	1.0 (6)	C2—C1—C8—C9	177.1 (3)

### Hydrogen-bond geometry (Å, º)

**Table:**

*D*—H···*A*	*D*—H	H···*A*	*D*···*A*	*D*—H···*A*
C1—O1···C9^i^		3.13 (1)		154 (1)
C8—H8*A*···O1^ii^	1.02 (4)	2.31 (4)	3.259 (5)	156 (3)
C7—H7···C10^iii^	1.06 (3)	2.71 (4)	3.626 (5)	144 (3)
C10—H10···Br1^iv^	0.93	2.68	3.305 (5)	126

Symmetry codes: (i) −*x*+1/2, *y*+1/2, −*z*+3/2; (ii) −*x*+3/2, *y*−1/2, −*z*+3/2; (iii) −*x*+1/2, *y*−1/2, −*z*+3/2; (iv) *x*−3/2, −*y*+1/2, *z*−1/2.
